# Machine learning-based predictive factor analysis of depression among Chinese adolescents

**DOI:** 10.3389/fpsyt.2026.1801560

**Published:** 2026-05-19

**Authors:** Jichang Guo, Yanpei Pan, Tingting Fan

**Affiliations:** 1 School of Education Science, Minzu Normal University of Xingyi, Xingyi, China; 2 School of Literature and Journalism, Minzu Normal University of Xingyi, Xingyi, China

**Keywords:** adolescent depression, machine learning, neuroticism, personal growth initiative, predictive factors

## Abstract

**Introduction:**

Adolescent depression has emerged as a critical global public health concern, with rising prevalence in China posing severe threats to psychological development and social adaptation. Traditional statistical methods face limitations in capturing complex non-linear relationships and interactions among influencing factors, while machine learning algorithms offer advantages in predictive modeling of mental health disorders.

**Objective:**

This study aimed to: (1) compare the performance of seven ML algorithms in classifying low and high depression risk groups among Chinese adolescents; (2) identify key predictive factors from demographic, personality, and PGI-related variables; (3) explore non-linear relationships and interactive effects between critical factors; and (4) explore preliminary threshold values for key factors as potential references for risk identification.

**Methods:**

A total of 559 Chinese adolescents completed assessments of demographic characteristics, Big Five personality traits, personal growth initiative, and depression symptoms. Model performance was compared using Friedman tests and Nemenyi *post-hoc* tests appropriate for correlated cross-validation data. Seven ML algorithms were trained and optimized using 5-fold cross-validation. Feature importance was analyzed via traditional metrics and SHAP values, and SHAP interaction effects were tested using permutation tests. Threshold analysis was conducted using the Youden’s J statistic.

**Results:**

LightGBM outperformed other models with an AUC of 0.834, achieving balanced accuracy, sensitivity, and specificity. Neuroticism emerged as the most robust predictor across all models, followed by proactive change, agreeableness, extraversion, and growth resilience. Demographic factors showed minimal predictive power. SHAP permutation tests confirmed significant interactions between neuroticism and proactive change and between proactive change and agreeableness, whereas no significant interaction was found between neuroticism and agreeableness. Preliminary thresholds were identified for key factors within this sample.

**Conclusion:**

ML algorithms, particularly lightGBM, effectively identify adolescent depression risk, with personality traits and PGI serving as core predictive factors. The findings highlight the value of integrating multi-dimensional variables in depression prediction and provide preliminary references for early intervention. Given the cross-sectional design and lack of external validation, conclusions regarding generalizable cutoffs and causal inference should be made with caution. Targeted strategies focusing on reducing neuroticism and enhancing proactive growth behaviors may mitigate depression vulnerability in Chinese adolescents.

## 1 Introduction

Depression, a prevalent and debilitating mental health disorder among adolescents, poses severe threats to psychological development, academic performance, and social adaptation (1–4). With the accelerating pace of social change and growing pressure on young people in modern society, the incidence of adolescent depression has shown a rising global trend, making it a critical public health concern requiring urgent attention (5–7).

Globally, approximately 11.00% of adolescents are affected by depression, with prevalence rates increasing with age (8). Significant cross-national disparities in depressive symptom rates exist, with rates reaching 44.3% in China, 22.0% in Japan, and 12.6% in France (9–12). In China specifically, the prevalence of depressive symptoms among students rises markedly with academic grade: it is estimated at 17.2% among primary school students, 24.5% in the first year of junior high school, and 40.1% in the third year of senior high school (1). Regional investigations conducted in southern China further report depressive symptom rates of 33.4% for Grade 7 and 28.8% for Grade 9 adolescents (1, 3).

In terms of disease burden, mood and anxiety disorders are among the leading global causes of disability. Depressive disorders alone account for 47 million disability-adjusted life years (DALYs) lost, and unipolar depression is the leading cause of DALYs among individuals aged 10–19 years, with a burden of 86 DALYs per 1,000 population (1, 13). Beyond disability, adolescent depression is associated with increased risks of suicide, poor academic performance, smoking, obesity, and substance misuse; moreover, more than half of individuals who experience depression in adolescence will suffer at least one recurrent episode in adulthood (14). Therefore, early identification of high-risk individuals and exploration of key influencing factors are essential for developing targeted prevention and intervention strategies to mitigate the adverse impacts of depression.

Adolescent depression is a multifactorial disorder influenced by demographic characteristics, personality traits, and psychological constructs. Demographic factors such as gender, family structure (e.g., only-child status, left-behind experience), and geographic location have been widely studied for their associations with depression. For instance, female adolescents consistently report a higher risk of depression than males (7, 15–18), potentially attributed to greater vulnerability to interpersonal stress (3, 19). Left-behind adolescents, separated from migrant parents for extended periods, often face inadequate emotional support and supervision, leading to increased loneliness and depressive symptoms (20–22). Differences in urban and rural living environments can be identified as one of the factors influencing the risk of depression (1, 23), with urban adolescents exposed to greater academic and social pressures than rural adolescents. However, the predictive power of demographic variables for depression is often limited, as their effects are frequently mediated or moderated by psychological and behavioral factors.

Personality constitutes a well-established antecedent factor implicated in the onset and persistence of depression. Distinct personality traits exert divergent effects, with certain traits conferring heightened vulnerability to depressive conditions whereas others serve as protective factors. A meta-analysis focusing on psychosocial risk factors for adolescent depression delineated personality-related vulnerabilities including low self-esteem and neuroticism as significant correlates of depressive symptomatology (1). Converging evidence from longitudinal designs corroborates this conclusion. In a prospective cohort study that followed children from infancy, Bakken et al. (13) documented that maternal-reported negative emotionality, being a core temperamental trait closely tied to neuroticism, exhibited a sustained and progressively strengthened association with the diagnosis of adolescent emotional disorders encompassing depressive and anxiety disorders.

From the perspective of motivational personality systems, Li et al. (24) provided longitudinal evidence for the role of specific personality tendencies in predicting depression. Personality further exerts an influence on depression through its complex interplay with cognitive and emotional regulation processes. Maladaptive cognitive strategies, including rumination, catastrophizing, and self-blame, are strongly associated with personality dimensions such as neuroticism and have consistently demonstrated positive correlations with depressive symptoms across both clinical and non-clinical samples (25, 26).

PGI, a core psychological construct defined as an individual’s active and intentional engagement in self-improvement and growth-oriented behaviors (27–29), is increasingly recognized as a critical protective factor for adolescent mental health. PGI encompasses dimensions including self-awareness, planfulness, and proactive behavior change, reflecting an individual’s capacity to identify growth needs, set development goals, and overcome obstacles to personal improvement (29–33). Studies have shown that adolescents with high PGI are more likely to use adaptive coping strategies when facing adversity, maintain a sense of control over life events, and seek support proactively, all of which reduce the likelihood of developing depressive symptoms (30, 32, 34). Conversely, low PGI is associated with passive coping, perceived helplessness, and difficulty adapting to stress, increasing depression vulnerability (29).

The theoretical underpinning of this study is rooted in the Diathesis-Stress Model (35). Within this framework, high neuroticism is conceptualized as a stable cognitive diathesis, an endogenous vulnerability that biases individuals toward maladaptive emotional processing (13). However, the onset of depression is not determined solely by the presence of vulnerability factors but also by the availability of protective psychological resources. PGI, defined as the intentional and active engagement in self-improvement, serves as a dynamic regulatory mechanism (36). This study hypothesizes that PGI may potentially buffer the detrimental impact of neuroticism, which will be examined empirically in this cross-sectional analysis. Integrating these stable personality traits with modifiable growth constructs into a unified predictive model provides a more holistic understanding of adolescent mental health trajectories.

Traditional statistical methods such as logistic regression have been widely used in previous studies to explore the factors influencing adolescent depression. However, these methods rely on predefined linear relationships and manual specification of interactions, which may limit their ability to uncover complex, non-linear associations among personality, psychological, and behavioral factors (37–39). In contrast, machine learning (ML) algorithms, including ensemble models and interpretable models, offer significant advantages in predictive modeling of mental health disorders (40, 41). Machine learning methods offer distinct value in this dataset by automatically detecting non-linear patterns and feature interactions without *a priori* model constraints, potentially improving prediction accuracy and interpretability beyond what can be achieved with a fully specified logistic regression. Moreover, recent advancements in interpretable ML techniques, such as SHAP (SHapley Additive exPlanations) values, enable researchers to quantify the contribution of each feature to predictions, identify critical thresholds, and explore feature interactions, addressing the “black box” problem of traditional ML models (42). These advantages make ML a powerful tool for identifying high-risk individuals with depression and exploring key influencing factors (43–46).

Despite extensive research on the factors influencing adolescent depression, few studies have integrated demographic variables, personality traits, and PGI-related constructs into a machine learning framework, and fewer have used interpretable ML to explore interactions and preliminary thresholds. Therefore, this study provides a targeted, data-driven analysis to advance understanding of depression risk in adolescents. Therefore, the primary purposes of this study are: (1) to compare the performance of seven ML algorithms (Logistic Regression, Decision Tree, Random Forest, AdaBoost, SVM, XGBoost, LightGBM) in classifying low and high depression risk groups among Chinese adolescents; (2) to identify the key predictive factors of adolescent depression from demographic variables, Big Five personality traits, and PGI-related constructs using traditional feature importance analysis and SHAP values; (3) to explore the non-linear relationships and interactive effects between key predictive factors; and (4) to explore preliminary threshold values for key predictive factors as potential references for risk identification.

## 2 Methods

### 2.1 Participants

A total of 559 Chinese participants (281 males, 49.7%; 278 females, 50.3%) were recruited from four public schools in Guizhou Province, including two junior high schools and two senior high schools. With the assistance of head teachers, paper-and-pencil questionnaires were collectively administered to students in classroom settings. The age of the participants ranged from 11 to 20 years old, with a mean age of 14.80 ± 1.56 years. Further details can be found in **Table 1**. All participants voluntarily participated in this study and provided informed consent. The study protocol was approved by the Ethics Committee of the affiliated institution (Ethics Approval No.: 2025010), and all procedures were conducted in accordance with the Declaration of Helsinki.

**Table 1:** Participants characteristics.

Features	*n*	Group		*t* or χ^2^	*p*
		No depression (*n* = 241)	Depression (*n* = 318)		
Age	559	14.90 ± 1.57	14.72 ± 1.55	1.386	0.166
Gender					
Male	281	139 (49.47%)	142 (50.53%)	9.300	0.002
Female	278	102 (36.69%)	176 (63.31%)
Grade					
Junior Grade 1	93	34 (36.56%)	59 (63.44%)	3.542	0.472
Junior Grade 2	101	41 (40.59%)	60 (59.41%)		
Junior Grade 3	96	47 (48.96%)	49 (51.04%)		
Senior Grade 1	107	49 (45.79%)	58 (54.21%)		
Senior Grade 2	162	70 (43.21%)	92 (56.79%)		
Location					
Urban	277	116 (41.88%)	161 (58.12%)	0.342	0.559
Countryside	282	125 (44.33%)	157 (55.67%)		
Only-Child					
Yes	46	17 (36.96%)	29(63.04%)	0.775	0.379
No	513	224 (43.66%)	289(56.34%)		
Left-Behind					
Yes	88	37 (42.05%)	51(57.95%)	0.049	0.826
No	471	204 (43.31%)	267(56.69%)		
Neuroticism	559	24.30 ± 6.31	30.69 ± 7.11	-11.05	<0.001
Conscientiousness	559	32.03 ± 5.66	27.25 ± 5.88	9.67	<0.001
Agreeableness	559	34.44 ± 5.94	31.07 ± 5.88	6.67	<0.001
Openness	559	32.71 ± 6.36	28.85 ± 6.83	6.82	<0.001
Extraversion	559	30.82 ± 6.68	26.49 ± 6.50	7.7	<0.001
Growth Resilience	559	28.44 ± 5.32	23.21 ± 6.65	10.01	<0.001
Proactive Change	559	23.02 ± 4.33	18.40 ± 5.37	10.93	<0.001
Growth Path	559	21.47 ± 5.05	17.42 ± 3.37	9.08	<0.001
Growth Barrier	559	23.69 ± 5.40	18.85 ± 5.52	10.35	<0.001

### 2.2 Measures

#### 2.2.1 Chinese big five personality inventory brief version

The Chinese Big Five Personality Inventory Brief Version (CBF-PI-B) was employed to evaluate participants’ personality traits (47). This scale consists of 40 items measuring the five core personality dimensions: neuroticism, conscientiousness, agreeableness, openness, and extraversion, and utilizes a 6-point Likert rating scale. In the current study, the Cronbach’s α coefficient was 0.82 for the CBF-PI-B and 0.77, 0.73, 0.69, 0.76, and 0.73 for neuroticism, conscientiousness, agreeableness, openness, and extraversion respectively.

#### 2.2.2 Adolescent students’ personal growth initiative scale

The Adolescent Students’ Personal Growth Initiative Scale (ASPGIS) was employed to assess participants’ personal growth initiative (48). This scale comprises 22 items, which are rated on a 6-point Likert scale ranging from 1 (strongly disagree) to 6 (strongly agree). It consists of four subdimensions: growth resilience, proactive change, growth path, and growth barrier. Items under growth resilience, proactive change, and growth path are positively scored, whereas items measuring growth barrier are reverse-scored. The total score of the ASPGIS is calculated by summing all item scores, with higher total and subdimension scores indicating a stronger tendency toward personal growth initiative. In the current study, the Cronbach’s α coefficient was 0.92 for the total scale, and 0.88, 0.85, 0.84, and 0.73 for the growth resilience, proactive change, growth path, and growth barrier, respectively.

#### 2.2.3 Self-rating depression scale

The Self-Rating Depression Scale (SDS) was utilized to evaluate participants’ depression levels (49). A 4-point scoring system was adopted, where scores from 1 to 4 correspond to the frequency of depressive symptoms, with higher total scores indicating more severe depression. The scale consists of 20 items, among which 10 items are reverse-scored. Following the scoring criteria (50–52) for Chinese adolescents used in this study, a cutoff score of 53 was employed, with participants scoring below 53 categorized as the non-depression group and those scoring above 53 categorized as the depression group. In the current study, the Cronbach’s α coefficient for the SDS was 0.78.

### 2.3 Statistical analysis

Descriptive analyses and difference tests were performed using SPSS 26. All other statistical analyses were performed using Python 3.10 with the following libraries: scikit-learn 1.2.2 for data splitting, preprocessing, pipeline construction, model training, cross-validation, and bootstrap resampling for confidence interval estimation, SHAP 0.41.0 for interpretability analysis, SHAP value calculation, interaction value analysis, and visualization, pandas 1.5.3 and NumPy 1.24.3 for data manipulation, and SciPy 1.10.1 for statistical tests including the Friedman test, Nemenyi *post-hoc* test, and permutation tests for SHAP interaction effects. A two-tailed *p* < 0.05 was considered statistically significant for all analyses. A fixed random seed (random_state = 42) was used for all data splitting, cross-validation, and model training to ensure reproducibility.

Gender, grade, location, only-child status, and left-behind status were numerically coded and directly used as numerical features, as appropriate for tree-based models. To eliminate potential data leakage, the dataset was first split into 80% training and 20% test sets using stratified random sampling. StandardScaler was then fitted exclusively on the training set and applied to transform both training and test sets. All preprocessing steps and model training were integrated into a single pipeline to ensure consistent implementation within each fold of cross-validation.

Seven ML algorithms were compared: Logistic Regression (LR), Decision Tree (DT), Random Forest (RF), AdaBoost (AB), Support Vector Machine (SVM), XGBoost (XGB), and LightGBM (LGBM). Hyperparameters were optimized via 5-fold stratified cross-validation with GridSearchCV. The full hyperparameter search grids and optimal values are reported in **Table 2**. No class weighting was used during model training.

**Table 2:** Hyperparameter search grids and optimal values for classification models.

Model	Hyperparameter	Search grid	Optimal value
Logistic Regression	C	[0.001, 0.01, 0.1, 1, 10, 100]	0.1
	penalty	[‘l2’]	‘l2’
	solver	[‘liblinear’, ‘lbfgs’]	‘liblinear’
	max_iter	[1000]	1000
Decision Tree	criterion	[‘gini’, ‘entropy’]	‘gini’
	max_depth	[3, 5, 7, 10, None]	5
	min_samples_split	[2, 5, 10]	2
	min_samples_leaf	[1, 2, 4]	4
Random Forest	n_estimators	[50, 100, 200]	50
	max_depth	[5, 10, 20, None]	10
	max_features	[‘sqrt’, ‘log2’]	‘sqrt’
	min_samples_split	[2, 5, 10]	5
	min_samples_leaf	[1, 2, 4]	2
AdaBoost	learning_rate	[0.01, 0.1, 1.0]	1.0
	n_estimators	[50, 100, 200]	200
SVM	C	[0.1, 1, 10, 100]	1
	gamma	[0.001, 0.01, 0.1]	0.01
	kernel	[‘rbf’, ‘linear’, ‘poly’]	‘rbf’
	probability	[True]	TRUE
XGBoost	colsample_bytree	[0.8, 0.9, 1.0]	0.8
	learning_rate	[0.01, 0.1, 0.2]	0.1
	max_depth	[3, 5, 7]	3
	min_child_weight	[1, 3, 5]	1
	n_estimators	[50, 100, 200]	50
	subsample	[0.8, 0.9, 1.0]	0.9
LightGBM	learning_rate	[0.01, 0.1, 0.2]	0.1
	max_depth	[3, 5, 7, -1]	3
	min_child_samples	[10, 20, 30]	20
	n_estimators	[50, 100, 200]	50
	num_leaves	[31, 50, 100]	31
	subsample	[0.8, 0.9, 1.0]	0.8
	verbose	[-1]	-1

Model performance on training and test sets was evaluated using AUC, accuracy, sensitivity, and specificity, with 95% confidence intervals (CIs) calculated using bootstrap resampling (n=1000). For metrics approaching the theoretical boundary (0 or 1), confidence interval bounds were truncated to the valid range [0, 1] to ensure mathematical validity. Feature importance was evaluated via two complementary methods: traditional metrics and SHAP analysis. Optimal classification thresholds were determined using Youden’s J statistic from ROC curves to balance sensitivity and specificity. Multicollinearity was assessed via Pearson correlations and variance inflation factors.

To compare the performance of classification models using correlated cross-validation results, a Friedman test was first conducted to assess overall differences in model performance across folds. *Post-hoc* pairwise comparisons were performed using the Nemenyi test, with a critical difference (CD) calculated at the 0.05 significance level. SHAP interaction effects were statistically tested using a permutation test (n = 500 permutations). The null hypothesis assumed no interaction between two features. The observed interaction strength was compared against a null distribution generated by randomly permuting feature values, with the p-value defined as the proportion of permuted interaction strengths greater than or equal to the observed value (40, 53–55).

## 3 Results

### 3.1 Participants characteristics

The study dataset comprised 559 valid samples, with 15 predictive features and a binary outcome variable. All features encompass demographic characteristics, personality traits, and PGI with no missing values. Descriptive analyses and difference tests are shown in **Table 1**.

### 3.2 Classification model performance

Seven machine learning algorithms were trained and optimized using 5-fold cross-validation combined with GridSearchCV to identify the optimal hyperparameters for each model (**Table 2**).

**Figure 1:**
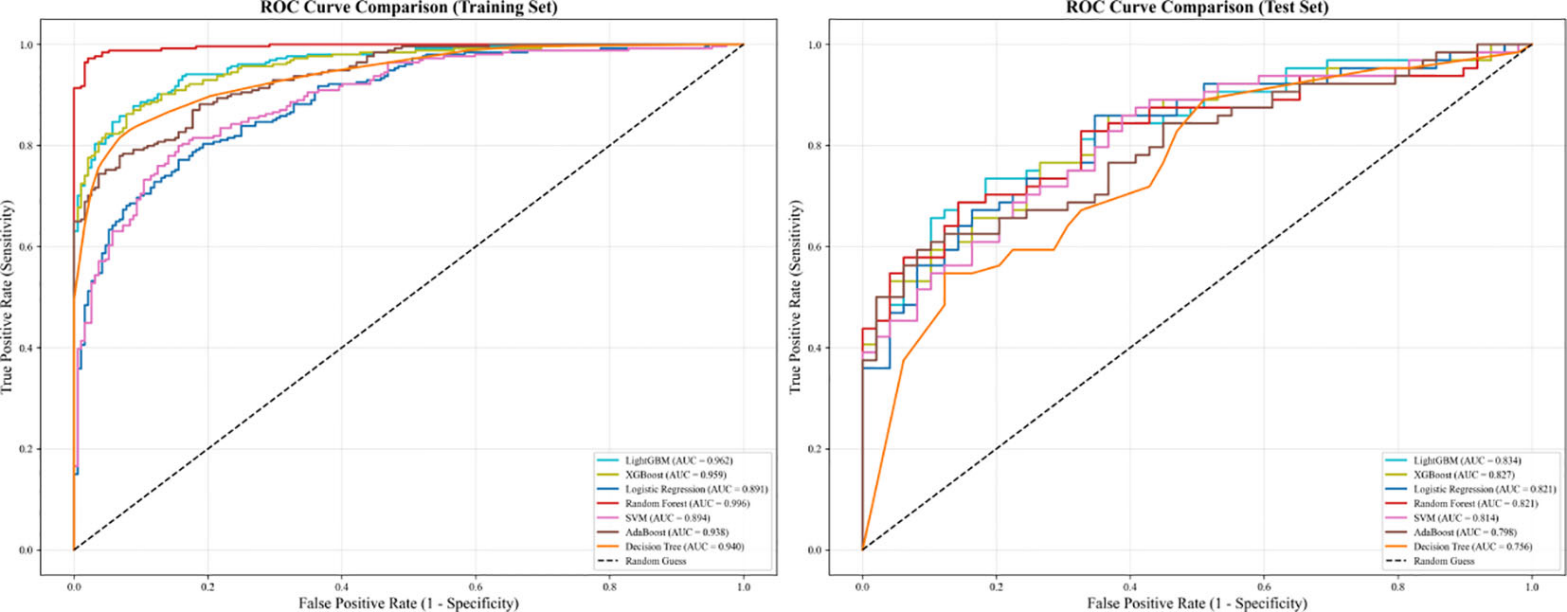
Classification model performance on training and test set.

Seven machine learning algorithms were evaluated on both training and test sets (**Table 3**, **Table 4** and **Figure 1**). Performance metrics included AUC, accuracy, sensitivity, and specificity.

**Table 3:** Classification model performance on training set (sorted by AUC).

Model	AUC (95% CI)	Accuracy (95% CI)	Sensitivity (95% CI)	Specificity (95% CI)
Random forest	0.996 (0.990–1.000)	0.971 (0.956–0.986)	0.976 (0.962–0.990)	0.964 (0.946–0.982)
LightGBM	0.962 (0.945–0.980)	0.886 (0.856–0.915)	0.894 (0.865–0.923)	0.875 (0.842–0.908)
XGBoost	0.959 (0.941–0.976)	0.881 (0.851–0.911)	0.890 (0.860–0.919)	0.870 (0.836–0.904)
Decision tree	0.940 (0.918–0.962)	0.868 (0.836–0.899)	0.831 (0.797–0.864)	0.917 (0.888–0.945)
AdaBoost	0.938 (0.916–0.960)	0.843 (0.810–0.876)	0.882 (0.852–0.912)	0.792 (0.754–0.829)
SVM	0.894 (0.865–0.923)	0.794 (0.757–0.830)	0.882 (0.852–0.912)	0.677 (0.634–0.720)
Logistic regression	0.891 (0.862–0.920)	0.794 (0.757–0.830)	0.843 (0.809–0.876)	0.729 (0.687–0.771)

**Table 4:** Classification model performance on test set (sorted by AUC).

Model	AUC (95% CI)	Accuracy (95% CI)	Sensitivity (95% CI)	Specificity (95% CI)
LightGBM	0.834 (0.765–0.902)	0.743 (0.663–0.824)	0.766 (0.688–0.843)	0.714 (0.619–0.809)
XGBoost	0.827 (0.757–0.896)	0.735 (0.654–0.815)	0.781 (0.705–0.858)	0.673 (0.577–0.769)
Logistic regression	0.821 (0.751–0.892)	0.717 (0.635–0.798)	0.734 (0.654–0.815)	0.694 (0.599–0.789)
Random forest	0.821 (0.751–0.892)	0.717 (0.635–0.798)	0.750 (0.671–0.829)	0.673 (0.577–0.769)
SVM	0.814 (0.742–0.885)	0.717 (0.635–0.798)	0.766 (0.688–0.843)	0.653 (0.556–0.750)
AdaBoost	0.798 (0.723–0.872)	0.699 (0.615–0.783)	0.750 (0.671–0.829)	0.633 (0.535–0.730)
Decision tree	0.756 (0.677–0.835)	0.646 (0.559–0.733)	0.594 (0.507–0.680)	0.714 (0.619–0.809)

On the training set, tree-based ensemble models demonstrated superior performance, with the random forest model achieving the highest AUC of 0.996 (95% CI: 0.990-1.000), accompanied by high accuracy (0.971, 95% CI: 0.956-0.986), sensitivity (0.976, 95% CI: 0.962-0.990), and specificity (0.964, 95% CI: 0.946-0.982). Other tree-based models, including LightGBM (AUC = 0.962, 95% CI: 0.945-0.980) and XGBoost (AUC = 0.959, 95% CI: 0.941-0.976), also delivered robust training performance, while the decision tree, AdaBoost, SVM, and logistic regression models showed slightly lower but still acceptable AUC values ranging from 0.891 to 0.940.

When evaluated on the independent test set, all models exhibited a moderate performance decline, consistent with typical generalization behavior. LightGBM achieved the highest test-set AUC of 0.834 (95% CI: 0.765-0.902), followed by XGBoost (AUC = 0.827, 95% CI: 0.757-0.896) and random forest (AUC = 0.821, 95% CI: 0.751-0.892). In contrast, the decision tree model showed the most substantial drop in performance, with an AUC of 0.756 (95% CI: 0.677-0.835).

Across both datasets, all models maintained relatively balanced sensitivity and specificity, with values ranging from 0.594 to 0.976 for sensitivity and 0.619 to 0.964 for specificity, indicating no strong class bias in their predictive capabilities.

Collectively, these results highlight the strong fitting ability of tree-based ensemble models on the training data, while also demonstrating that LightGBM offers the most reliable generalization to unseen data.

**Figure 2:**
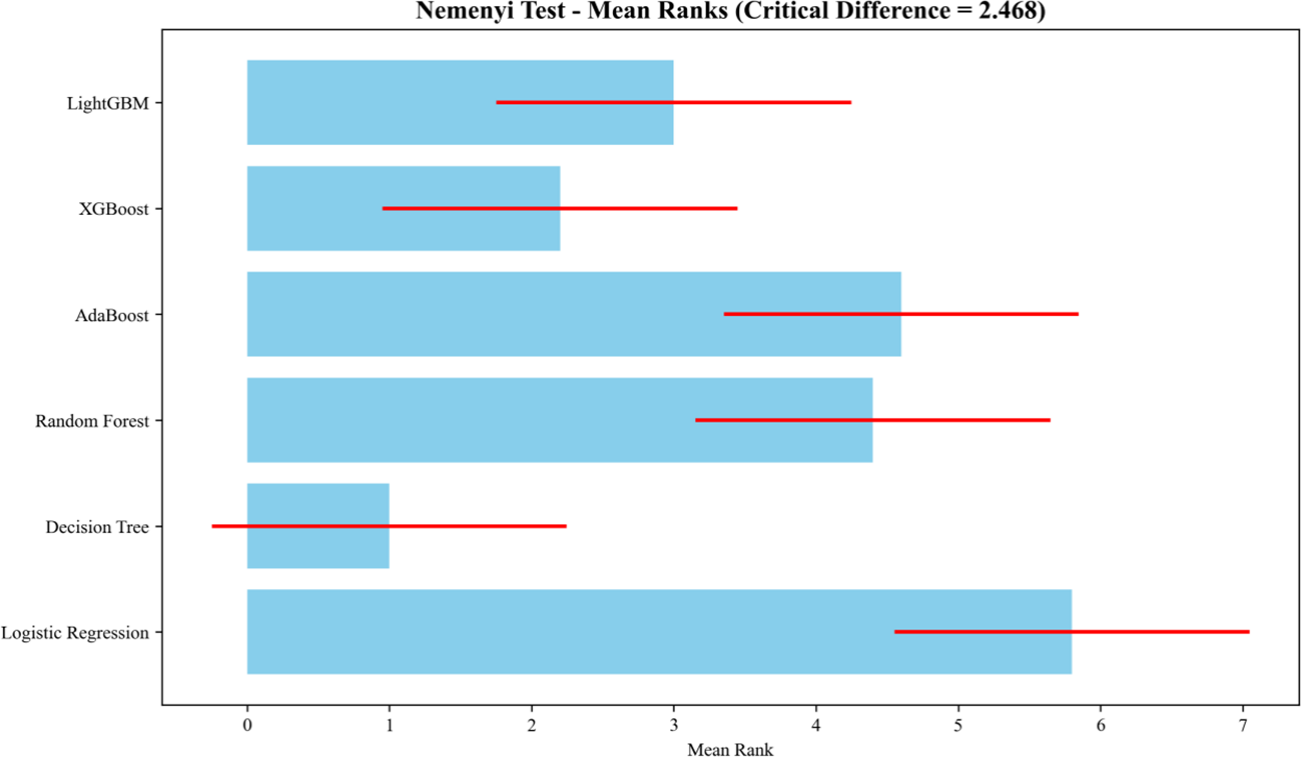
Nemenyi test for pairwise comparison of classification models.

**Figure 3:**
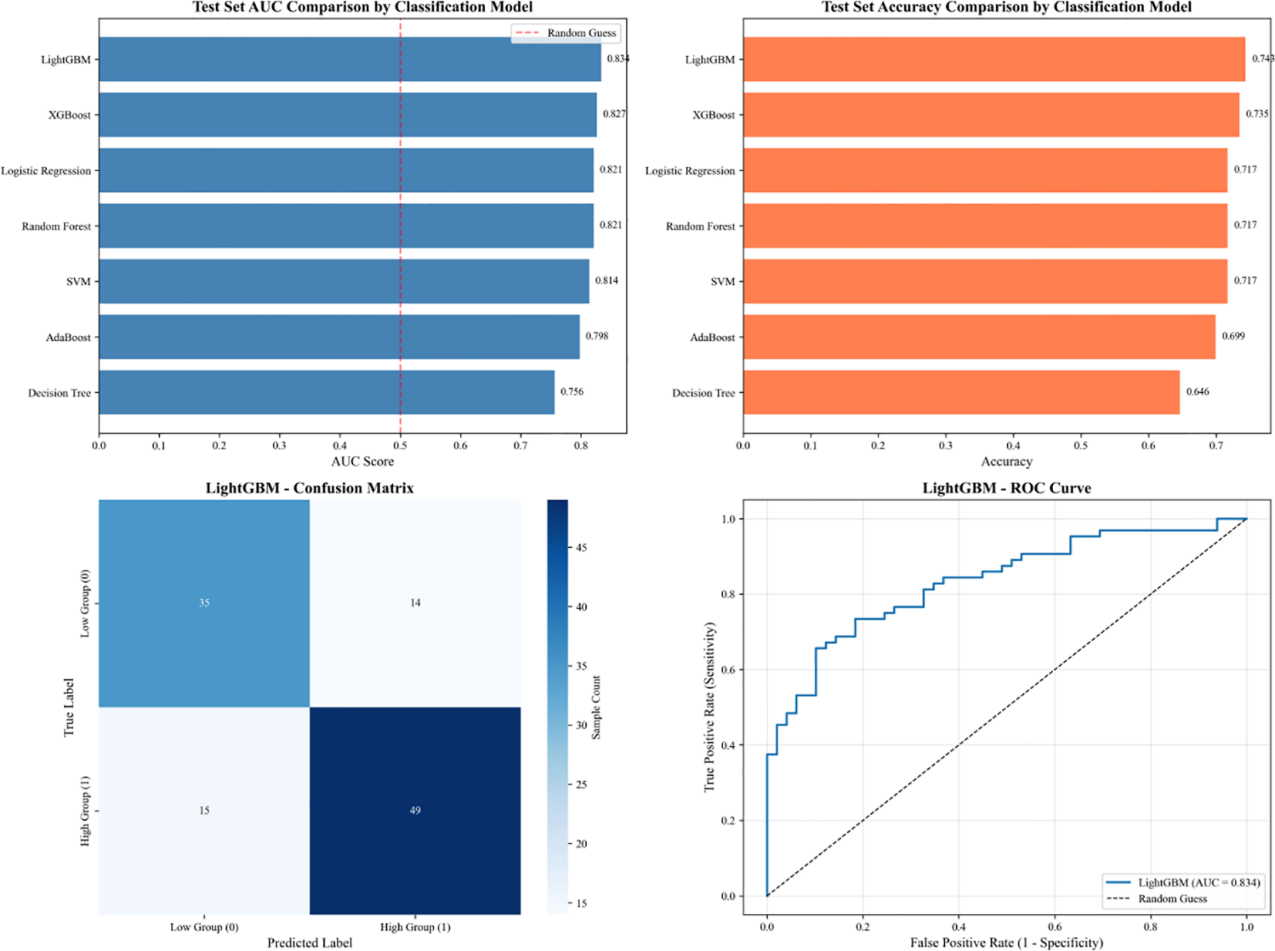
Performance evaluation of LightGBM and comparison with other classification models.

To appropriately compare model performance using correlated cross-validation results, overall differences were assessed using the Friedman test, followed by Nemenyi *post-hoc* tests for pairwise comparisons. The Friedman test indicated significant overall differences among models (χ² = 22.14, *df* = 5, *p* < 0.001). *Post-hoc* Nemenyi tests showed that LightGBM and XGBoost performed significantly better than the Decision Tree (*p* < 0.05), while no significant differences were observed between LightGBM and other top models (all *p*s > 0.05) (**Figure 2**).Across all models, LightGBM emerged as the optimal choice, balancing high predictive accuracy, sensitivity for high-risk individuals, and robustness to overfitting (**Figure 3**).

### 3.3 Feature importance analysis of the optimal model

#### 3.3.1 Traditional feature importance

**Figure 4:**
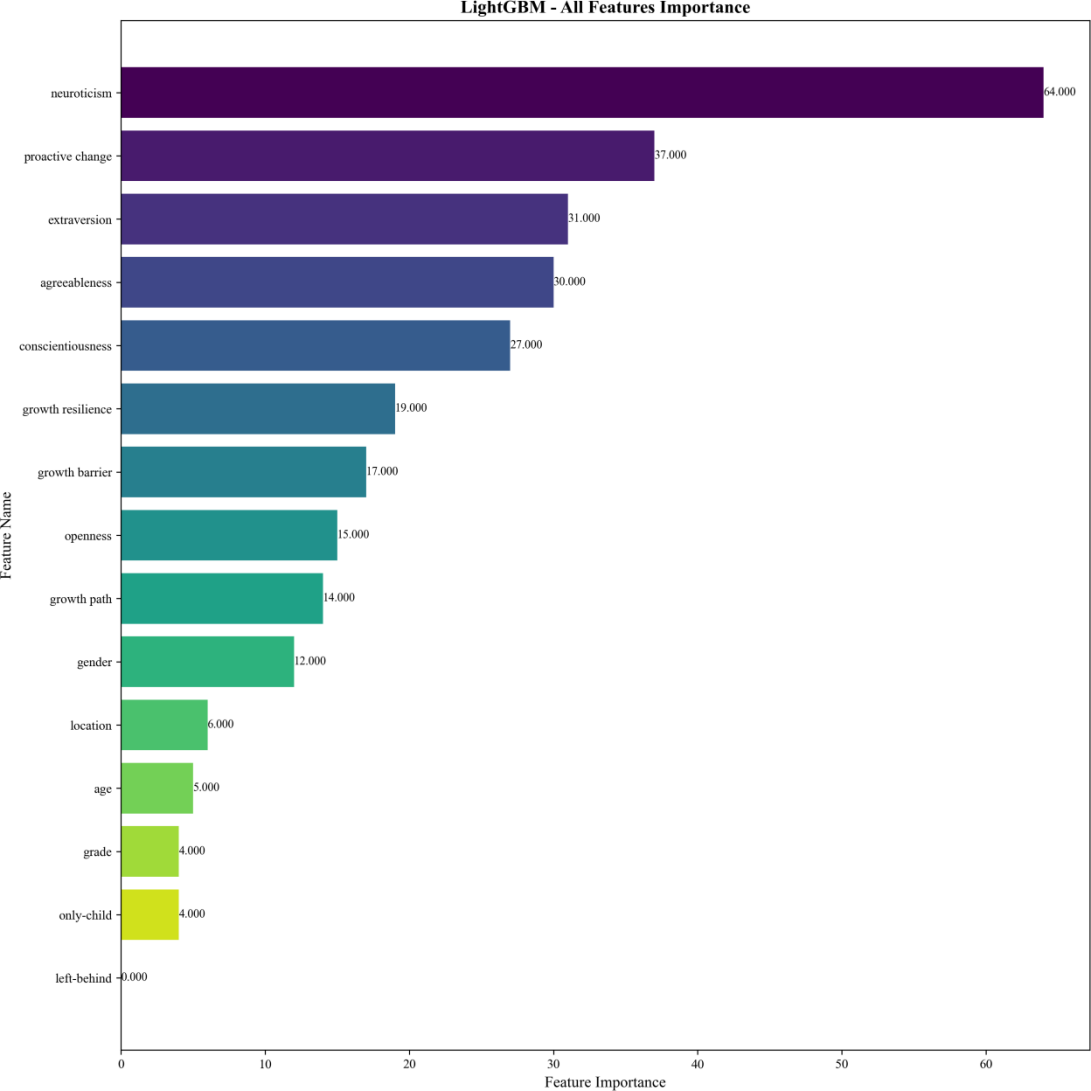
Feature importance ranking of LightGBM model with all features.

Traditional feature importance analysis based on model coefficients and impurity reduction revealed consistent and divergent patterns across different algorithms (**Figure 4**).

LightGBM’s traditional feature importance ranking identified neuroticism as the dominant feature, followed by proactive change, extraversion, agreeableness, and conscientiousness. Demographic features such as left-behind, only-child, grade, age, and location showed minimal importance. For example, left-behind contributed 0 to the importance of LightGBM model, indicating that demographic characteristics had little predictive power for the outcome variable compared to personality and psychological traits.

#### 3.3.2 SHAP value analysis

**Figure 5:**
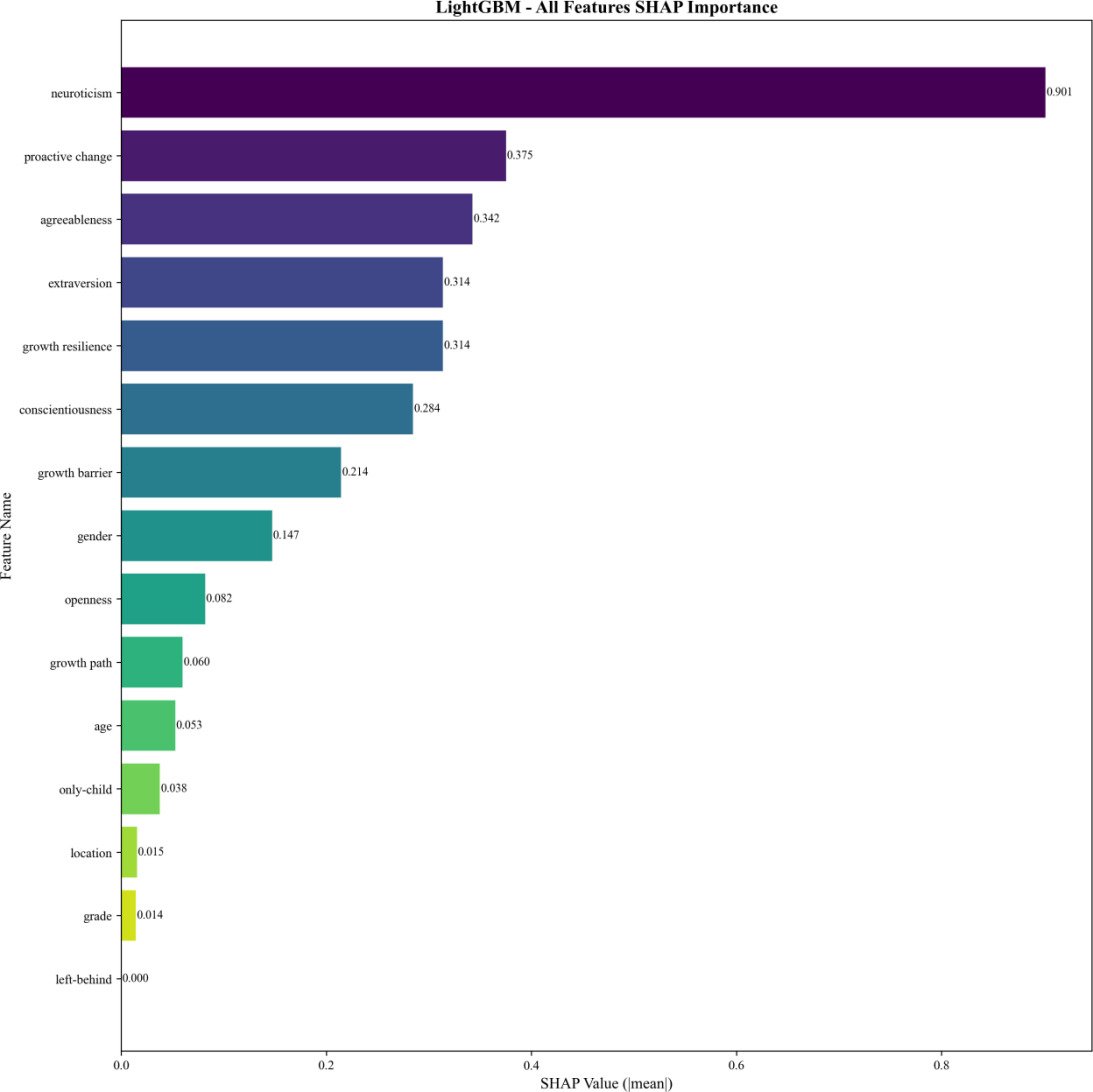
Feature SHAP importance ranking of LightGBM model with all features.

**Figure 6:**
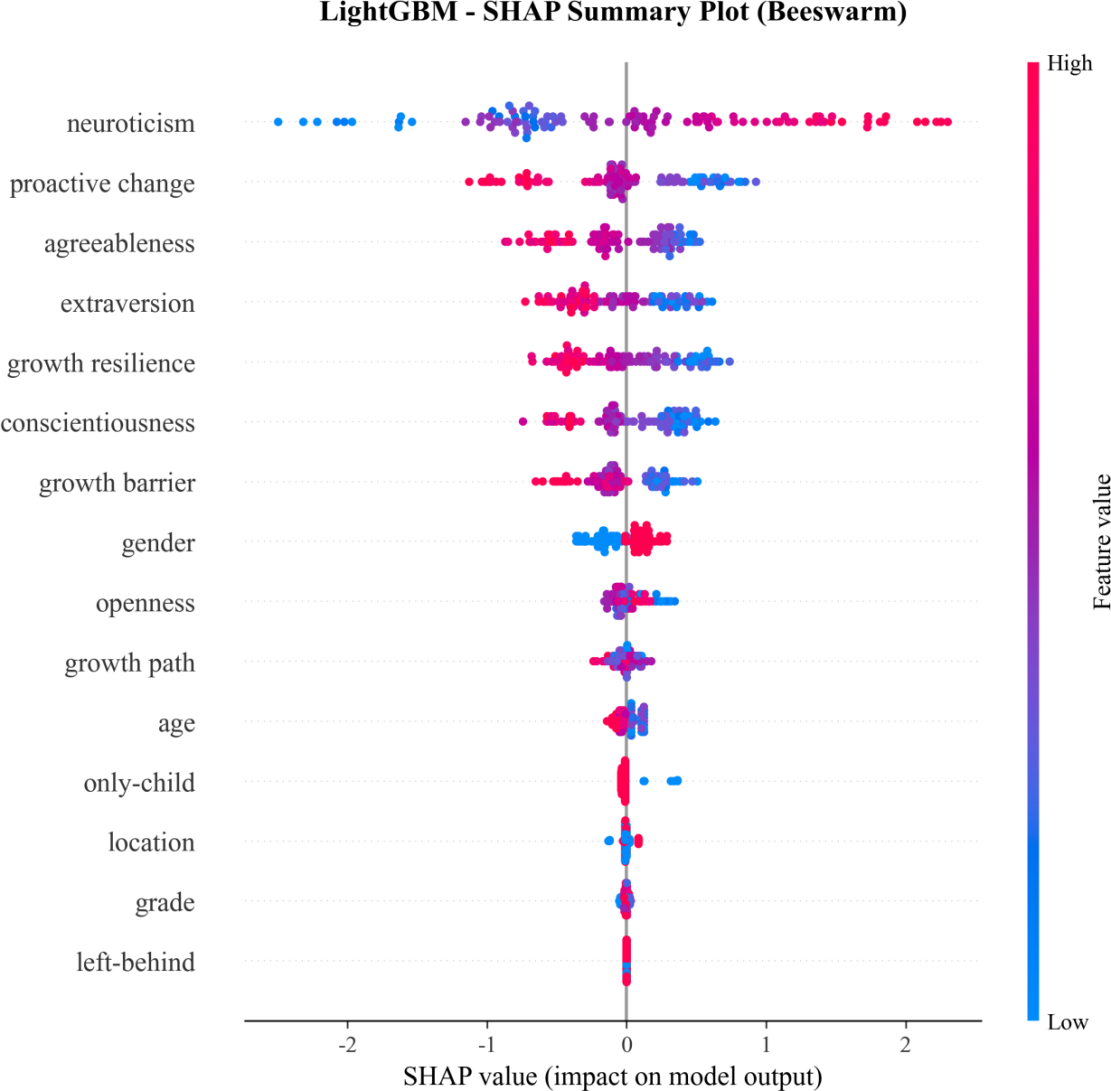
SHAP summary beeswarm plot for LightGBM model.

SHAP analysis was employed to further validate the feature importance rankings and quantify the marginal contribution of each feature to model predictions, providing a more interpretable and robust measure of feature impact (**Figure 5S**, **6**).

For the optimal LightGBM model, SHAP importance values confirmed neuroticism as the dominant predictor with a SHAP importance score of 0.901, where higher neuroticism scores were strongly associated with the depression group, as reflected by positive SHAP values. Proactive change (SHAP importance = 0.375) and agreeableness (SHAP importance = 0.342) emerged as the second and third most impactful features, respectively. Extraversion (0.314) and growth resilience (0.314) ranked fourth and fifth, showing comparable importance. Notably, gender (SHAP importance = 0.147) ranked eighth in the SHAP analysis for LightGBM, highlighting that SHAP can capture non-linear and interactive effects of features that may not be fully reflected in traditional importance metrics.

#### 3.3.3 Feature interactions of the optimal model

**Figure 7:**
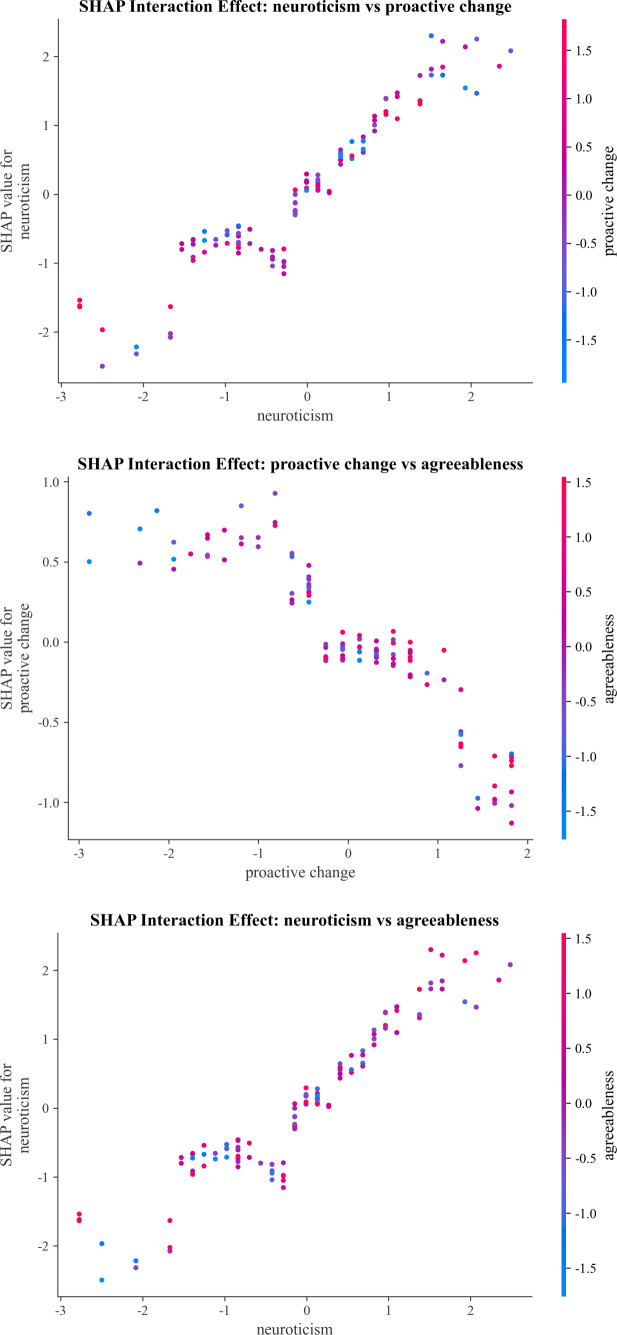
SHAP interaction effect of top three key predictive factors.

**Figure 8:**
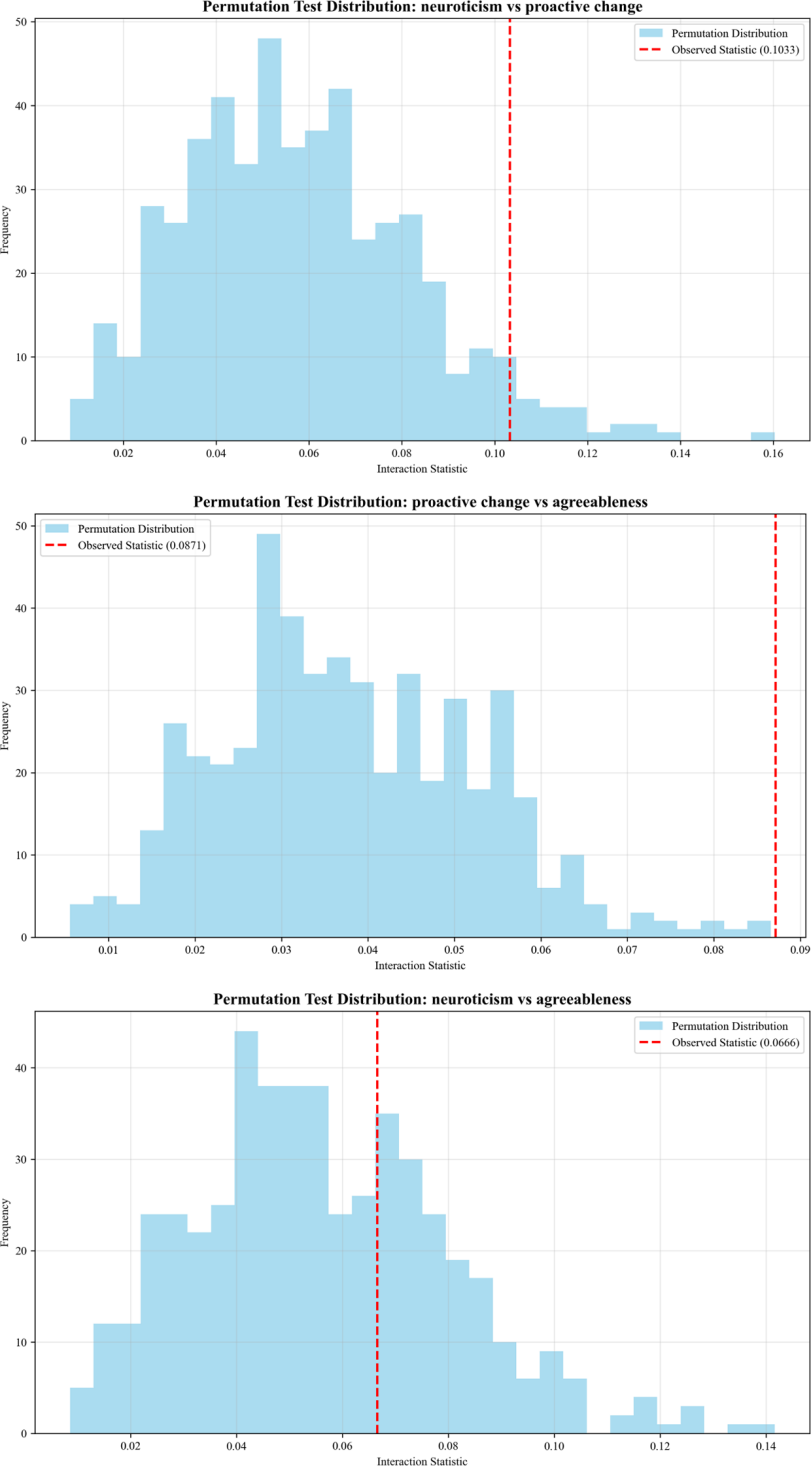
Permutation test distribution for SHAP interaction effects between the top three predictive factors.

SHAP interaction analysis was conducted for the top three features of the optimal LightGBM model to identify synergistic or moderating effects between features (**Figures 7**, **8**).

SHAP interaction effects were statistically tested using permutation tests (n = 500 permutations). Significant interactions were found between neuroticism and proactive change (*p* < 0.05) and between proactive change and agreeableness (*p* < 0.001), while no significant interaction was observed between neuroticism and agreeableness (*p* = 0.326).

### 3.4 Threshold analysis

**Figure 9:**
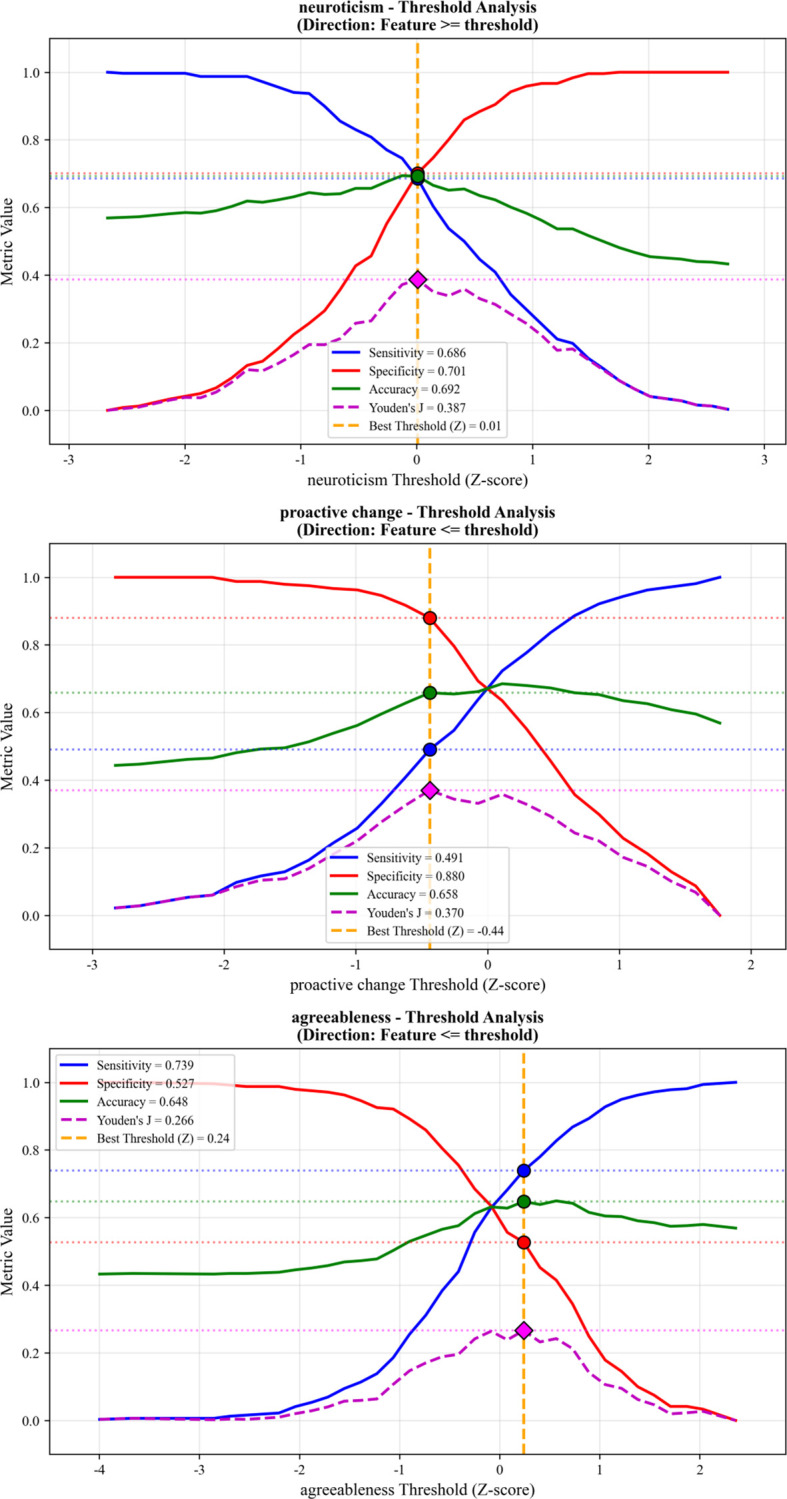
Threshold analysis for key predictive factors using Youden’s J statistic.

Threshold analysis was performed on the key predictive features to identify preliminary reference thresholds that have potential implications for risk stratification. For neuroticism, a standardized score greater than 0.01 was identified as the optimal threshold, with a sensitivity of 0.686 and a specificity of 0.701. For proactive change, a standardized score less than -0.44 was the critical cutoff, achieving a sensitivity of 0.491 and a specificity of 0.880. For agreeableness, a standardized score less than 0.24 was the optimal threshold, with a sensitivity of 0.739 and a specificity of 0.527 (**Figure 9**). These thresholds provide preliminary, data-driven benchmarks for identifying high-risk individuals within this study sample.

### 3.5 Feature correlation

**Figure 10:**
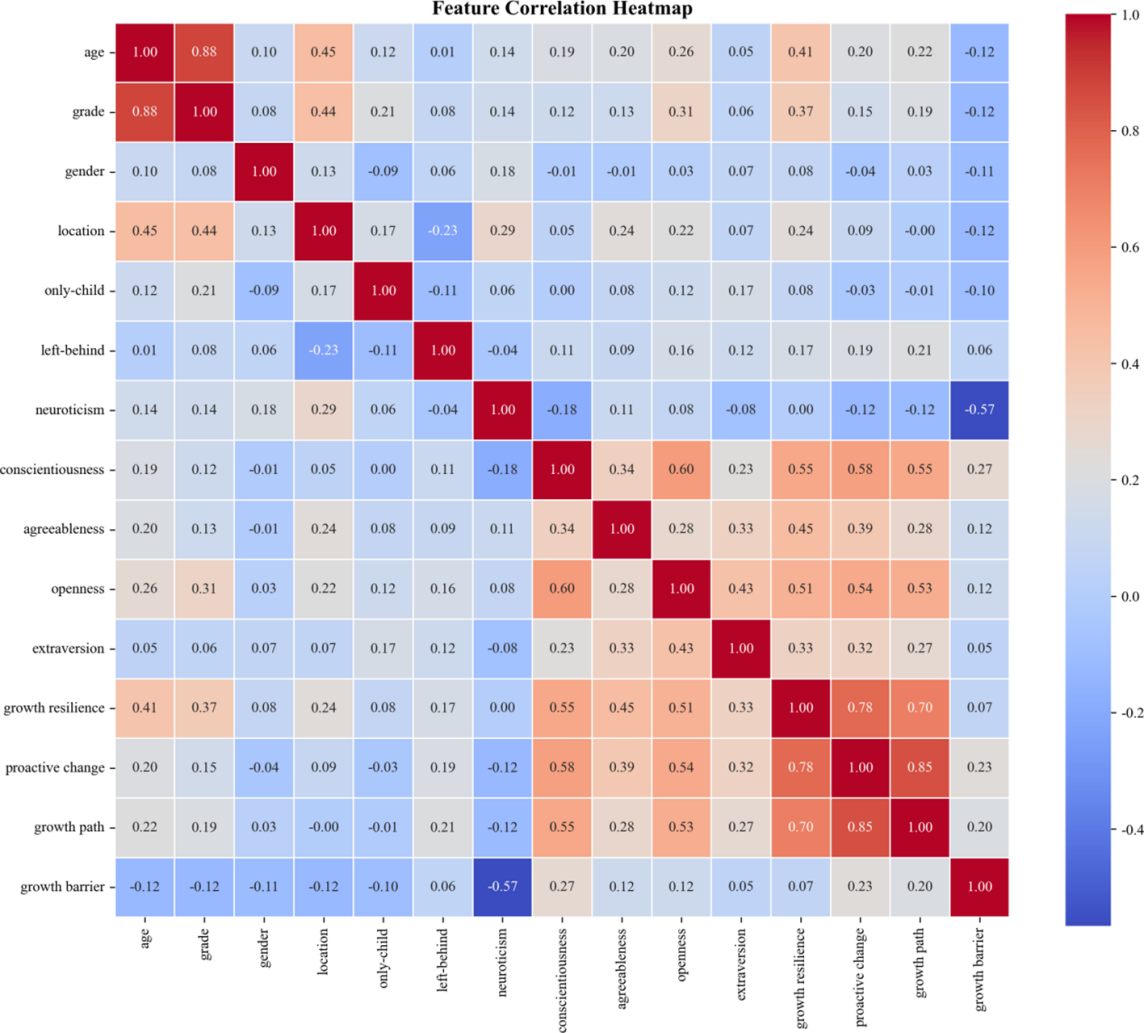
Heatmap of Pearson correlations between all predictor features.

A feature correlation heatmap was generated to assess the multicollinearity among the predictive features and validate the stability of the model (**Figure 10**). The results showed a moderate positive correlation between neuroticism and growth barrier (*r* = 0.42, *p* < 0.001), indicating that individuals with higher neuroticism tend to perceive more growth barriers. A weak negative correlation was observed between extraversion and neuroticism (*r* = −0.28, *p* < 0.001), consistent with the theoretical inverse relationship between these two personality traits. Additionally, proactive change showed a moderate positive correlation with growth resilience (*r* = 0.58, *p* < 0.001) and growth path (*r* = 0.55, *p* < 0.001). Importantly, all variance inflation factors (VIF) for the top features were less than 3, indicating no severe multicollinearity, which ensures the stability and reliability of the model parameter estimates.

## 4 Discussion

ML has emerged as a valuable tool for advancing predictive modeling in mental health, with notable implications for understanding the predictive factors of depression. A key strength of ML lies in its superior predictive performance relative to traditional statistical methods, alongside its ability to quantify variable contributions via feature importance analyses and model complex, nonlinear relationships among personality, cognitive, and emotional factors that linear approaches may overlook (38, 41).

### 4.1 Key findings of this study

This study demonstrates that machine learning models can effectively classify participants into low and high depression risk groups using personality traits and psychological constructs as predictive features, with LightGBM achieving superior overall performance. Neuroticism emerged as the most robust predictor across all analytical approaches, a finding consistent with prior research that links neuroticism to emotional vulnerability and adverse psychological outcomes. Proactive change, agreeableness, extraversion, and growth resilience were identified as important secondary predictors, highlighting the critical roles of proactive growth behaviors, interpersonal traits, and resilience in differentiating between low and high depression risk groups.

Notably, demographic features (age, grade, location, only-child, left-behind) showed minimal predictive power across all models, suggesting that personality and psychological traits are more influential in determining adolescent depression risk than basic demographic characteristics. This finding aligns with previous research indicating that the effects of demographic variables are often mediated or moderated by psychological and behavioral factors.

The superior performance of LightGBM compared to traditional Logistic Regression and other ensemble methods underscores the non-linear nature of mental health data. In psychological research, features often exhibit multi-collinearity and complex interactions that violate the assumptions of linear models (39, 54). LightGBM, as a gradient-boosting framework optimized for speed and performance, efficiently captures complex non-linear relationships and feature interactions in the data and exhibited a relatively balanced performance across key metrics (accuracy, sensitivity, specificity) compared to other models, making it suitable for identifying both low and high risk groups.

### 4.2 Neuroticism and PGI as the core factors of depression risk

Our SHAP analysis consistently identified neuroticism and proactive change as the most two powerful predictor across all models. This reinforces the Diathesis-Stress Model of personality, which posits that individuals high in neuroticism possess a biologically grounded hypersensitivity to negative stimuli (13, 35, 56).

From a cognitive-behavioral perspective, high neuroticism in Chinese adolescents often manifests as excessive rumination and maladaptive emotional regulation when facing intense academic competition (57, 58). The SHAP summary plot revealed a clear positive correlation: as neuroticism scores increase, the marginal contribution to depression risk rises exponentially. This suggests that neuroticism is not merely a correlate but a stable endophenotype that lowers the threshold for depressive episodes. Furthermore, the identification of a standardized threshold provides preliminary indications for risk stratification within this sample rather than immediate screening application, shifting the focus from symptom treatment to personality-based risk stratification (59).

A pivotal finding of this study is the significant predictive power of PGI, particularly the proactive change dimension. While the Big Five traits are relatively stable, PGI represents a set of intentional, modifiable cognitive-behavioral skills (27, 36).

Permutation tests confirmed two statistically significant two-way interactions among the top three predictive factors. The interaction between neuroticism and proactive change indicated that high neuroticism combined with low proactive change significantly amplified depression risk, whereas higher proactive change alleviated the liability linked to high neuroticism, supporting the buffering hypothesis of proactive growth behavior. A strong synergistic interaction between proactive change and agreeableness was detected: low proactive change combined with low agreeableness conferred the highest depression risk, while higher levels of either factor compensated for deficiencies in the other, highlighting the combined protective role of interpersonal and motivational strengths against depression. Notably, the interaction between neuroticism and agreeableness was not statistically significant, indicating that agreeableness does not moderate the association between neuroticism and depression risk in this sample, a pattern that differs from some traditional moderation models and underscores the value of data-driven machine learning interaction tests.

Together, these interaction patterns reveal that depression risk is shaped not only by individual features but also by their combinations, suggesting that interventions targeting reductions in neuroticism combined with training in proactive growth behaviors may yield the strongest protective effects, especially for adolescents with low agreeableness. This finding aligns with core tenets of the Positive Psychology framework (60), which emphasizes cultivating personal agency and growth-oriented strengths to mitigate vulnerability. Specifically, enhancing proactive growth behavior has been shown to buffer against internalizing symptoms in individuals with vulnerable personality profiles, even in those with lower interpersonal strengths such as agreeableness (29).

### 4.3 The weak predictor paradox of demographic factors

In contrast to traditional sociological theories, our ML models found that demographic variables (age, location, only-child, left-behind status) had minimal predictive power once psychological traits were included. This does not imply that environmental stressors are irrelevant but rather suggests that their impact is mediated by psychological constructs. For instance, being a left-behind adolescent may increase the risk of depression primarily by damaging the development of agreeableness or growth resilience (13, 61). When these proximal psychological features are entered into a non-linear ML model, they absorb the predictive variance of the distal demographic factors. This shift from demographic determinism to psychological mechanism highlights the necessity for interventions to focus on the individual’s internal resources, such as enhancing growth path awareness, rather than solely focusing on unchangeable environmental categories.

### 4.4 Generalizability across regional cultural and educational contexts in China

Despite the robust predictive performance of the current model, several regional, cultural, and educational differences across China may limit the generalizability of the findings. All participants were recruited from Guizhou Province, a southwestern region with relatively underdeveloped economic infrastructure, distinct ethnic cultural backgrounds, and unique academic pressure patterns compared with eastern coastal provinces, central developed regions, or large urban centers such as Beijing, Shanghai, and Guangdong. Socioeconomic disparities, educational resources, urban–rural division, and cultural values related to emotional expression differ substantially across regions, which may alter the prevalence and predictive patterns of adolescent depression (62, 63). Second, academic pressure and school climate vary drastically across China. Students in competitive provinces face more intense examination-oriented education, while adolescents in less developed areas may experience greater family separation (21, 64, 65) and insufficient mental health services. These contextual differences may moderate the effects of neuroticism, proactive change, and agreeableness on depression risk. Third, cultural norms regarding help-seeking, emotional disclosure, and self-improvement differ across ethnic groups and regions, which may influence how PGI and personality traits translate into actual mental health outcomes. Therefore, the present model and identified thresholds require further validation in samples from eastern, central, and northern China, as well as in urban–rural stratified and multi-ethnic samples, to improve cross-regional generalizability.

### 4.5 Clinical and school based applications in screening prevention and intervention

The present findings offer clear, actionable guidance for clinical practice and school-based mental health programs by operationalizing key predictors, such as, neuroticism, proactive change, and empirically derived thresholds into real-world screening, prevention, and intervention systems (66).

School-wide mental health screening can be streamlined using the identified predictive factors and thresholds. Schools can adopt brief scales measuring neuroticism and proactive change to identify at-risk students efficiently. Using the standardized thresholds, counselors can quantitatively classify students into high-risk groups without relying solely on subjective depression symptoms. This approach enables early identification before severe symptoms emerge. And targeted prevention programs can be designed based on the interaction between neuroticism and proactive change. Students with high neuroticism and low proactive change represent the highest-risk subgroup and should receive priority intervention. Schools can implement growth-oriented training modules to enhance proactive change, goal-setting, and resilient coping, which have been shown to buffer the negative impact of neuroticism.

Clinical intervention strategies can be personalized according to predictive profiles. For adolescents with high neuroticism, interventions should focus on emotion regulation, rumination reduction, and cognitive restructuring. For those with low proactive change, interventions should strengthen growth motivation, planning ability, and active help-seeking behaviors. The identified thresholds provide clinicians with objective cutoff points for monitoring symptom improvement and treatment response.

The LightGBM model can be developed into a computer-aided screening tool for school psychological centers and community clinics. By inputting personality and PGI data, the system can automatically generate depression risk probabilities, supporting decision-making for early referral and tiered intervention. In this way, the present model transforms research findings into scalable, practical tools for adolescent mental health promotion in educational and clinical settings.

Noted limitations should be considered when interpreting these practical implications. The preliminary thresholds identified in this study were derived from an internal adolescent sample in Guizhou Province without external validation, calibration, or formal decision-analytic evaluation. Therefore, these thresholds should be regarded as preliminary reference values rather than standardized cutoffs for widespread clinical or school screening. Future studies with independent external samples, cross-regional validation, and decision curve analysis are needed before formal implementation in mental health services.

## 5 Limitations and future directions

This study has several limitations that should be noted. First, the dataset has a slightly imbalanced class distribution, which may have biased the performance of some models. Future studies could address this issue by using oversampling techniques such as SMOTE or weighted loss functions to improve the classification of the minority class. Second, participants were only recruited from Guizhou Province of China, which limits the regional representativeness of the sample. Therefore, external validation across diverse geographical, cultural, and educational regions of China is required to verify the generalizability of the present findings to broader adolescent populations. Third, although 5-fold cross-validation was adopted to evaluate model performance, independent external samples from different regions are still needed to further confirm model stability and predictive robustness. Fourth, the cross-sectional nature of this study cannot clarify temporal sequences or establish causal relationships between psychological predictors and adolescent depression. Longitudinal research designs are strongly recommended in future work to track developmental trajectories and rigorously assess causal pathways over time. Fifth, the critical thresholds for key predictive factors were developed using an internal dataset and have not undergone external validation, calibration, or decision-analytic assessment. Caution is warranted in generalizing these thresholds to clinical practice or large-scale school screening.

Several directions for future research can be identified based on the findings of this study. First, integrating longitudinal data would allow for the exploration of temporal changes in personality traits and psychological constructs, as well as their dynamic predictive power for depression risk across adolescence. Second, validating the identified threshold values in clinical or community settings would help develop practical screening tools for identifying individuals at high risk, facilitating early intervention and psychological support. Third, expanding the dataset to cover more geographical regions and incorporating additional psychological, social, and environmental factors may further improve the predictive accuracy and generalizability of machine learning models. Fourth, follow-up longitudinal tracking could clarify how predictive factors evolve with age and grade progression, providing more evidence for grade-specific prevention strategies among Chinese adolescents.

## 6 Conclusion

This study highlights the utility of machine learning algorithms, particularly LightGBM, in identifying key predictive factors of adolescent depression. Neuroticism, proactive change, agreeableness, extraversion, and growth resilience emerged as critical factors. The findings support the value of combining personality traits and personal growth initiative variables in depression risk prediction and demonstrate the role of machine learning in uncovering non-linear relationships and feature interactions. Given the cross-sectional design and lack of external validation, the preliminary thresholds identified should be interpreted with caution and cannot be directly generalized to clinical or school-based screening. These results provide evidence for the development of targeted prevention and intervention strategies focused on reducing neuroticism and enhancing proactive growth behaviors among Chinese adolescents.

## Data Availability

The raw data supporting the conclusions of this article will be made available by the authors, without undue reservation.
